# Mediterranean Diet Information on TikTok and Implications for Digital Health Promotion Research: Social Media Content Analysis

**DOI:** 10.2196/51094

**Published:** 2024-06-19

**Authors:** Margaret Raber, Haley Allen, Sophia Huang, Maria Vazquez, Echo Warner, Debbe Thompson

**Affiliations:** 1 Department of Health Disparities Research MD Anderson Cancer Center Houston, TX United States; 2 Department of Kinesiology Rice University Houston, TX United States; 3 Baylor College of Medicine Houston, TX United States; 4 The University of Utah Huntsman Cancer Institute Salt Lake City, UT United States; 5 Baylor College of Medicine Children's Nutrition Research Center Houston, TX United States

**Keywords:** misinformation, social media, Mediterranean Diet, content analysis, health communication, communication, TikTok, diet, cardiometabolic disease, cardiometabolic, consumer, eating, social media, quality, mHealth, mobile health, digital health, promotion research, nutrition therapy, healthy diet

## Abstract

**Background:**

The Mediterranean diet has been linked to reduced risk for several cardiometabolic diseases. The lack of a clear definition of the Mediterranean diet in the scientific literature and the documented proliferation of nutrition misinformation on the internet suggest the potential for confusion among consumers seeking web-based Mediterranean diet information.

**Objective:**

We conducted a social media content analysis of information about the Mediterranean diet on the influential social media platform, TikTok, to examine public discourse about the diet and identify potential areas of misinformation. We then analyzed these findings in the context of health promotion to identify potential challenges and opportunities for the use of TikTok in promoting the Mediterranean diet for healthy living.

**Methods:**

The first-appearing 202 TikTok posts that resulted from a search of the hashtag #mediterraneandiet were downloaded and qualitatively examined. Post features and characteristics, poster information, and engagement metrics were extracted and synthesized across posts. Posts were categorized as those created by health professionals and those created by nonhealth professionals based on poster-reported credentials. In addition to descriptive statistics of the entire sample, we compared posts created by professionals and nonprofessionals for content using chi-square tests.

**Results:**

TikTok posts varied in content, but posts that were developed by health professionals versus nonprofessionals were more likely to offer a definition of the Mediterranean diet (16/106, 15.1% vs 2/96, 2.1%; *P*=.001), use scientific citations to support claims (26/106, 24.5% vs 0/96, 0%; *P*<.001), and discuss specific nutrients (33/106, 31.1% vs 6/96, 6.3%; *P*<.001) and diseases related to the diet (27/106, 25.5% vs 5/96, 5.2%; *P*<.001) compared to posts created by nonhealth professionals.

**Conclusions:**

Social media holds promise as a venue to promote the Mediterranean diet, but the variability in information found in this study highlights the need to create clear definitions about the diet and its components when developing Mediterranean diet interventions that use new media structures.

## Introduction

Social media is an increasingly important resource for health information, including content related to nutrition [1,2]. Over 72% of American internet users reported looking for health information on the web in 2013 [3]. Additional global data offers insight into current trends, with 93% (n=192) of young urban adults in Ghana [4] and 85% (n=42,113) of French adults [1] reporting using the internet or social media when seeking nutrition information. Additionally, over one-third of respondents in a nationally representative survey reported learning to cook from web-based resources such as blogs and instructional videos [5], demonstrating the multiple ways social media can impact the home food environment and consumer knowledge and beliefs.

Social media offers a powerful platform to communicate dietary recommendations and health promotion materials; however, poor information quality and misinformation (eg, health or nutrition claims that are incorrect, exaggerated, inappropriately extrapolated, and misleading or otherwise contrary to current scientific evidence) undermine these efforts by creating consumer confusion [6]. Web-based recipes and diet information content are not required to include accurate nutritional labeling or to qualify health claims [7]. Previous surveillance of food and nutrition content has shown overall poor nutritional quality of web-based recipes and high levels of inaccurate health claims [8,9]. Information in this domain exists on a spectrum from high-quality scientifically accurate resources to extreme fad diets lacking support from scientific research and potentially harmful nutritional supplements [2,7,8]. Qualitative approaches to targeted surveillance are needed to understand web-based nutrition information and identify potential barriers to the effective use of social media for public health nutrition efforts.

Dietary recommendations for disease prevention have shifted over the last decade from a focus on macro and micronutrients to food-based dietary patterns [10]. The Mediterranean diet (MedDiet; broadly defined as rich in produce, whole grains, and moderate in poultry and fish) has emerged as an increasingly popular dietary pattern for cardiovascular disease prevention [11]. Currently recommended by several heart health organizations, multiple studies have established associations between higher adherence to the MedDiet and reduction in total mortality, as well as an inverse association with death due to coronary heart, vascular, cancer, and neurodegenerative diseases [12-24].

Promoting the MedDiet in the United States may reduce cardiovascular disease incidence and mortality, but relatively few Americans adhere to the MedDiet [25,26]. A widely accepted definition of what the MedDiet entails is lacking, creating opportunities for confusion among US consumers about what the MedDiet is and how to follow it [14,27,28]. The US Community Preventive Services Task Force recommends digital interventions as a cost-effective strategy to improve dietary intake and reduce nutrition-related disease [29]. Social media holds promise as a venue to deliver nutrition intervention content, but little is known about how the MedDiet is portrayed on the web. Social media–based health interventions do not occur in a vacuum, as intervention participants are inevitably concurrently exposed to a broader swath of web-based information. Without a better understanding of the scope, characteristics, and content of MedDiet information on social media, efforts to use social media for MedDiet promotion are limited.

The objective of this study was to describe public discourse about the MedDiet on the influential social media platform, TikTok. By conducting this study, we sought to answer the research question: “What are the characteristics, scope, and content of MedDiet-related information a user may encounter on TikTok?” This study offers insight into potential areas of confusion or misinformation on this topic that may deter from factual health messaging and undermine health promotion efforts.

## Methods

### Study Design

We conducted a social media content analysis on information labeled with the hashtag #mediterraneandiet on TikTok. TikTok offers a platform for users to create, disseminate, and share information via continuous streams of curated short-form videos. TikTok is currently one of the most downloaded nongaming apps. Worldwide, TikTok has accrued over 2 billion downloads and retains 1 billion active monthly users, with 130 million users in the United States [30]. TikTok has recently been leveraged for health promotion [31], including efforts to highlight the importance of mask-wearing during the COVID-19 pandemic [32] and education on common dermatologic conditions [33]. Food- and diet-related posts on TikTok are common, with #mediterraneandiet having 77.6 million views as of August 2022. Other food trends such as #ketodiet and #veganrecipes are even more prolific, with 1.9 billion and 2.9 billion views, respectively (assessed as of August 2022). TikTok was the data source selected for this study given its extensive reach and the popularity of food-related content on the platform, suggesting its immense potential for wide-reaching health education efforts. The specific hashtag “#mediterraneandiet” was used for this study as it was the most popular tag related to the health topic of interest, the MedDiet.

In August 2021, two independent coders created unique study accounts on TikTok and each downloaded the first-appearing 200 TikTok videos that resulted from a search of the hashtag #mediterraneandiet. As TikTok promotes content based on an algorithm that factors in the reach of the post itself (views, likes, comments, and shares), the popularity of the content creator (followers, engagement, and sponsorship), the geographic location of the user, and previous engagement with similar content, 2 separate new accounts were used to generate a prevaried sample. This approach was used to simulate a new user’s experience exploring the hashtag. The unit of analysis for the study was each individual video post. Following the approach of other social media studies [8,34], each row in the data set represented a unique video post, and data were quality checked (eg, completeness and duplication) prior to coding. Downloads of the videos as well as links to the videos on TikTok were retained in the data set to enhance data quality and ensure completeness if included posts were later altered or deleted. Of the 400 videos identified by the 2 coders, 99.5% (n=398) were duplicates (only 2 were found by only 1 coder) for a final sample of 202 videos. This high percentage of overlap in the first-appearing 200 videos identified on the 2 study accounts suggests the final sample includes the majority of videos a new user would encounter when exploring the #mediterraneandiet hashtag.

Each downloaded TikTok video in the final sample (n=202) was coded for 62 variables across four main categories, including (1) account details of the creator (number of followers, type of account, external links on account page, inclusion of product or service promotions, health related, or other professional credentials), (2) engagement metrics (number of post likes, shares, and comments), (3) post characteristics (host characteristics, setting, style, structure, use of text, humor, music, graphics, main message, and inclusion of promotions or product sales), and (4) post content specific to the MedDiet (definition of the MedDiet, foods discussed or displayed, discussion of nutrients, calories, diseases or disease risk factors related to the MedDiet, and use of scientific sources). Codes and corresponding definitions were maintained in a codebook adapted from previous studies examining the characteristics of TikTok content [32,33,35] and nutrition information on other social media platforms [8,9]. The codebook was refined by the research team using an iterative inductive-deductive process adapted from interview-based qualitative research [36]. The final codebook and categorization of variables are available in Multimedia Appendix 1. Two independent coders applied the first draft of the codebook to 15 videos from the sample, and new codes were added to capture additional information deemed to be important to the research question (eg, specific MedDiet foods promoted). Additional codes were then reviewed, collapsed, or expanded through group discussion with the 2 coders and the first (MR) and senior (DT) author. The updated codebook was then applied to 3 videos by 3 independent coders, discrepancies and inconsistencies were discussed and resolved by the research team, and the codebook was finalized. Two coders then double-coded 20% (40 videos) of the sample using the finalized codebook. Coder results were compared. Interrater reliability was examined using Cohen κ and was found to be high (k=0.843), indicating “near perfect” agreement [37]. The 2 coders then independently applied the codebook to all remaining videos.

Each variable was summarized to describe the MedDiet content on TikTok. Descriptive statistics including means, ranges, and SDs were applied to engagement metrics; content metrics were examined using frequencies and percentages. We evaluated differences in MedDiet variables by creator type (health professionals and nonhealth professionals) using chi-square tests. All statistics were completed using SPSS (version 23; IBM).

### Ethical Considerations

Data obtained from TikTok were publicly available; therefore, review by the institutional review board was not completed. Creators of the data (posts) used in this study data are not identified in this publication; however, the full data set of posts is available to researchers upon reasonable request to the first author.

## Results

A total of 202 unique TikTok video posts were included in this analysis. Select post creator information, characteristics, and engagement metrics are shown in Table 1. Regarding account details of creators, a large portion of content creators claimed to be health professionals on their profile page (n=106, 52.2%). Several account profiles included external links and 40 (19.8%) were promoting specific services or products on their profile pages.

**Table 1:** Select characteristics extracted from TikTok posts (n=202) using #mediterraneandiet.

Characteristics		Values
**Creator type,** **n (%)**		
	Health professional	106 (52.5)
	Nonhealth professional	96 (47.5)
**Creator profile characteristics, n (%)**		
	Profile contains external links	161 (79.7)
	Profile promotes products or services	40 (19.8)
**Post characteristics, n (%)**		
	Host speaking to the camera	100 (49.5)
	Food only (no host)	98 (48.5)
	Response to comment	63 (31.2)
	Recipe	62 (30.7)
	Health-related primary message	157 (77.7)
	Mediterranean culture’s primary message	45 (22.3)
Length (seconds), mean (SD)		39.6 (31.8)
**Post engagement metrics^a^, mean (SD)**		
	Likes	10,116.0 (56,548.3)
	Comments	134.0 (462.8)
	Shares	514.0 (3952.5)

^a^Engagement metrics not adjusted for the length of time posted on the site.

The 202 included posts were styled either a single host talking directly to the camera (n=100, 49.5%) or images of food or food preparation with no clear host (n=98, 48.5%). Relatedly, almost a third (n=62, 30.7%) of posts were recipes. Approximately one-third of videos (n=63, 31.2%) were formatted as a response to a comment, wherein the creator “responds” to a comment that another user wrote on a previous post. Responses to comments were typically structured as replies to requests for information, rebuttals of incorrect information, or opportunities to build discourse by extending content discussions. Other hashtags on included posts were extracted; the 10 most common co-occurring hashtags were #weightloss (n=51, 25.2%), #doctorsoftiktok (n=39, 19.3%), #fyp (n=39, 19.3%), #foodtiktok (n=26, 12.9%), #weightlossjourney (n=22, 10.9%), #food (n=22, 10.9%), #culinarymedicine (n=20, 9.9%), #foryou (n=18, 8.9%), #diet (n=17, 8.4%), and #doctor (n=16, 7.9%).

The 202 videos tagged with #mediterraneandiet fell into 1 of 2 main groups regarding its primary message; 157 (77.7%) of videos were identified as health focused, meaning the content related to health and healthy eating in some way, while 45 (22.3%) of videos were culture focused, meaning the content had no connection to health, instead focusing exclusively on the culture of regions near the Mediterranean Sea (eg, Greek Hotels and Italian restaurants).

Post content specific to the MedDiet was examined. The promotion of key components of the MedDiet (eg, fish, olive oil, and fruit or vegetables) varied in the sample (Table 2). The majority of culture-focused videos (31/45, 69%) and some health-focused videos (16/157, 10%) promoted non-MedDiet foods such as red meat, sweets, refined grains, and processed foods. Overall, the majority of videos (n=165, 81.6%) included at least 1 core component of the MedDiet with fruit and vegetables being the most common and fish, poultry, and whole grains being less common. While there were some differences between health-focused and culture-focused videos, clear examples emerged of individuals conflating these 2 concepts, with some users actively endorsing the MedDiet for health, while promoting regional foods that are not considered part of the MedDiet (ie, lamb gyro and baklava).

**Table 2:** Promotion of MedDiet^a^ and non-MedDiet foods in reviewed posts by health or culture focus.

Food	Total posts (n=202), n (%)	Health-focused posts (n=157), n (%)	Culture-focused posts (n=45), n (%)
Non-MedDiet foods	47 (23.3)	16 (10.2)	31 (68.9)
Fish	36 (17.8)	32 (20.4)	4 (8.9)
Olive Oil	54 (26.7)	40 (25.5)	14 (31.1)
Fruit or vegetables	138 (68.7)	105 (67.3)	33 (73.3)
Whole grains	42 (20.8)	40 (25.5)	2 (4.4)
Poultry	30 (14.9)	20 (12.7)	10 (22.2)
Nuts or beans	56 (27.7)	48 (30.6)	8 (17.8)

^a^MedDiet: Mediterranean diet.

The level of detail about the MedDiet varied by creator type (Table 3). While 84 (41.6%) of the 202 videos included a general claim that the MedDiet was healthy and 52 (25.7%) noted that the MedDiet is recommended by doctors, only 18 (8.9%) offered any description of what the MedDiet entails and relatively few (n=26, 12.9%) referenced scientific sources for health claims. Videos that were created by health professionals, defined as those that claim some health credential on their profile page, were more likely to offer a definition of the MedDiet (16/106, 15.1% vs 2/96, 2.1%; *P*=.001), discuss specific nutrients (33/106, 31.1% vs 6/96, 6.3%; *P*<.001) and diseases (27/106, 25.5% vs 5/96, 5.2%; *P*<.001) related to the MedDiet, and use scientific citations to support claims (26/106, 24.5% vs 0/96, 0%; *P*<.001) compared to those that did not claim to be health professionals.

**Table 3:** Mediterranean diet (MedDiet) information in posts on TikTok that use #mediterreaneandiet created by health professionals versus nonprofessionals.

Video content variable	Health professionals (n=106), n (%)	Nonprofessionals (n=96), n (%)	Pearson chi-square (*df*=1)	Cramer V	*P* value
Promote any MedDiet foods (Table 2)	83 (78.3)	82 (85.4)	1.70	0.092	.21
Mention the MedDiet as healthy generally	57 (53.8)	27 (28.1)	13.64	0.260	<.001
Mention the MedDiet is recommended by doctors	48 (45.3)	4 (4.2)	44.55	0.470	<.001
Mention any nutrients	33 (31.1)	6 (6.3)	20.02	0.315	<.001
Mention any diseases	27 (25.5)	5 (5.2)	15.52	0.277	<.001
Mention studies related to the MedDiet	26 (24.5)	0 (0)	27.03	0.366	<.001
Offer a description of the MedDiet	16 (15.1)	2 (2.1)	10.37	0.227	.001

Status as a health professional was indicated directly in videos either by statements or professional signifiers such as scrubs, white coats, or hospital badges. Of the 106 videos created by health professionals (as indicated on their account page), fewer than half gave their credentials (n=50, 47.2%) or had professional signifiers (n=27, 25%) present in the videos. All videos in which the host claimed to be a health professional or included a professional signifier were created by those claiming to be health professionals on their account page.

## Discussion

This study offers a glimpse at potential content that TikTok users may encounter when seeking MedDiet information on the web. Overall, our findings suggest that social media users will find some high-quality content created by health professionals, as well as some conflicting or vague information when exploring the #mediterraneandiet on TikTok.

Social media has emerged as an important resource for healthy lifestyle information and offers a plethora of nutrition content from practical recipes to detailed health information posts [9,38,39]. The structure of social media supports a connectivist approach to nutrition education [40], wherein individuals use multiple sources to continuously procure and provide information in a collaborative process [41]. This approach to acquiring nutrition information is a deviation from traditional models of patient health education and creates an environment wherein information sources can be legitimized, even without appropriate experience or credentials. As such, the participation of qualified professionals who provide high-quality information on social media is an important aspect of reducing the impact of misinformation. Promoting accurate information before exposure to misinformation is a recommended strategy for reducing the impact of health misinformation [6]. Our findings indicate that videos created by those who claim to be health professionals were more likely to discuss nutrients, disease prevention, existing scientific studies, and the MedDiet as a concept compared to videos created by nonprofessionals. These findings are in line with Kong et al [39], who found diabetes-related posts on TikTok varied in information quality depending on the creator. Other studies exploring different health topics on TikTok including dermatology [33] and vaccination [42] similarly found that creators claiming health credentials produced higher-quality educational content with more accurate information. Given these findings, health professionals such as nutritionists may consider exploring and identifying social media accounts or creators that offer high-quality content, in order to help support patients in navigating digital spaces related to MedDiet information.

Users may face potential difficulties when trying to determine the source of MedDiet information on social media. In the current analysis, fewer than half (50/106, 47.2%) of the videos in this sample made by health professionals included any verbal or visual indication of their credentials. Because TikTok is structured to be viewed as a continuous stream of videos, a user would have to backtrack into an account page to identify the credentials of a creator. This disrupts the continuous stream of content that is a signature of the TikTok platform and may be particularly ill-suited to these types of social media structures, where videos are typically under 45 seconds long. In the context of health promotion efforts, intervention and public messaging content created for social media may consider using signifiers of health professionalism such as badges, institutional insignia, or medical attire to reinforce trust in messages that are integrated into continuous video streams.

Our findings indicated that 45 (22.3%) of the 202 videos were unrelated to health, focusing exclusively on Mediterranean culture. The majority of these “culture” posts promoted foods that were not aligned with the MedDiet as a healthy eating pattern, such as red meat, sweets, refined carbohydrates, and processed foods. This finding illustrates a disconnect on social media between the MedDiet as a healthy eating pattern and the cuisines of Mediterranean countries. While the MedDiet is inspired by traditional cuisines from the Mediterranean, it represents an idealized diet of the region and does not include all foods that are currently eaten in Mediterranean countries or those served at Mediterranean restaurants [14,27]. This conflation of concepts creates the potential for confusion about what the MedDiet entails. As an example, red meat is eaten in Mediterranean countries but is distinctly not promoted as part of the MedDiet eating pattern. Additionally, consumers considering a shift to the MedDiet eating pattern may be discouraged by the notion that it would require a dramatic change to their current eating style or cultural food norms. Confusion about dietary recommendations [2,43] and mismatch between recommendations and existing food norms [44,45] reduce consumer confidence and are documented barriers to healthy eating. As such, interventions to promote the MedDiet in diverse settings may include information on how the MedDiet can be interpreted through different cuisines and tailor MedDiet messaging to the cultural food norms of target intervention groups.

The effect of exposure to nutrition information on social media on dietary intake is not well understood. Previous research suggests higher digital or electronic health literacy (one’s ability to effectively find, assess, and apply health information on the web) [46] is associated with some healthier diet and exercise behaviors [47,48], but research is limited. Additionally, understanding one’s ability to process web-based health information is distinct from measuring exposure to misinformation about specific topics.

While this study offers insight into the information a TikTok user would encounter on the web, it does not examine what a user does with that information. Future studies may explore the relationship between content exposure and home food behaviors, although that was outside the scope of this paper. Additionally, this study is limited by the use of a single hashtag; restriction of posts to those available in English; and use of a single social media platform, TikTok. The reliance on hand-coding limited our ability to include large numbers of posts, but the study sample of 202 is in line with previous studies qualitatively examining social media content related to nutrition [8]. Future studies could consider building on this qualitative work to train large language models and other artificial intelligence tools to code larger numbers of posts from across different sites. Another limitation of this work is that we did not attempt to individually verify the health credentials of creators or quantitatively score the scientific accuracy of the health information presented.

This content analysis is strengthened by using a systematic approach for identifying and analyzing social media posts from TikTok, including the use of 2 independent coders. Additionally, this is the first study to examine web-based MedDiet information, a critical topic given the popularity of using the internet for identifying nutrition information and the growing body of literature supporting MedDiet for health. Although the sample is not large (n=202) relative to the number of total posts on the topic, the high number of duplicates identified by 2 TikTok accounts indicates that the selected posts are representative of what a new account would generate at that point in time under the #mediterraneandiet.

These results have implications for health promotion professionals. While the MedDiet has been heralded as a dietary pattern that extends significant health benefits, this study highlights several challenges to implementation. Thus, it is important that health care providers clearly define the MedDiet when making recommendations, acknowledge the issue of varying information quality, and support the dissemination of accurate scientific content. The conflation of the MedDiet with Mediterranean cuisine found in this study emphasizes the need for individualized counseling and tailored interventions to adapt key components of the MedDiet to fit individual and community food preferences, sociocultural norms, and access.

Social media holds great promise to deliver public health interventions to a wide audience [31], but the structure of social media also lends itself to the creation and promotion of information that may be misleading. Future research is needed to develop and test the efficacy of digital MedDiet interventions in improving diet and knowledge while dispelling misinformation. Integrated tools such as labeling verified health professional accounts or enhancing quality assessments of health content may help individuals navigate these new types of media. Additionally, more research on the effect of nutrition misinformation exposure on dietary intake will be critical to identifying key intervention target populations and determining the efficacy of misinformation intervention tools.

## Appendix

Multimedia Appendix 1 Codebook variables and definitions.

## Abbreviations

MedDiet: Mediterranean diet

## Data Availability

The data sets generated and analyzed during this study are available from the corresponding author on reasonable request.
