# Supplementary material for: Mediterranean Diet Information on TikTok and Implications for Digital Health Promotion Research: Social Media Content Analysis
Source: JMIR Form Res. 2024 Jun 19;8:e51094. doi: 10.2196/51094 (PMC11222766; doi:10.2196/51094)
Supplement: Multimedia Appendix 1 [file formative_v8i1e51094_app1.pdf]

Category	Variable name	Code	Definition
Account details of creator	num_followers	Integer	Number of followers of Tiktok account from which recipe was posted (middle number under handle)
	handle	Text	Copy and paste Tiktok handle
	name	Text	Poster name if available (top of page)
	tagline	Text	Description of Tiktok account- do not include links or emoji
	account_type	Text	Type of account creator that made the post (examples: individual, sponsored, science communicators, non-profit, government, business, other
	profile_Link	1=yes, 0=no	If there is a hyperlink included on the profile page of poster
	Tag_Link_Text	Text	Add hyperlink name if available
	Tag_link_type	Text	Type of site the link from the tagline takes you to (youtube video, personal blog, amazon shopping site, etc)
	Tag_Link_Hyperlink	Text	hyperlink
	Tag_Link_Type_NEW	Text	topical blog, recipe site, product/retail/services (non-clinical), clinical services, social
	Tag_Link_Product_Service	1=yes, 0=no	product is sold on front page or link to product being sold on front page
	Tag_Link_Type_code	1=topical blog 2=recipe site 3=product/retail/services (non-clinical) 4= clinical services 5=social	code for tag_link_type_new
	account_type_NEW	1= clinician 2=RD 3= other health pro 4= individual 5=business 6=food professional 7=brand	Type of account creator that made the post either clinician 1, RD 2, other health pro 3, individual 4, business 5, food professional 6, brand 7
	account_type_healthpro	1=yes, 0=no	account creator claims to be some form of health professional
Engagement Metrics	likes	Integer	Number of likes
	comments	Integer	Number of comments
	shares	Integer	Number of shares
	Other_tags	Text	hashtags other than #mediterraneandiet separated by commas
Post Characteristics	length	Text	Length of video (seconds)
	invideo_credentials	0=none, 1= health professional, 2=food professional	Host verbally or in accompanying text claims to be a healthcare or food professional. If the only indication is in the handle (i.e. @doctormd or @superfoodie) that does NOT count. The host needs to SAY or SHOW in TEXT that they are a health or food professional.
	professional_signifier	0=none / plain dress, 1= healthcare (scrubs, white coat, hospital insignia), 2= foodpro (chef's hat or coat, restaurant insignia)	Host is wearing clothing that indicates a healthcare or food profession
	gender	1=female, 2=male, 3=both, 4= other, etc	Gender of person in video, select the most likely. For example, if a person is in a costume, select the most likely gender of the person in the costume (not the gender of the costume subject) SELECT ONE ONLY
	age	1= child/teen, 2=adult under 45, 3=adult +45	Apparent age of person in video, use multiple if several different people of different age ranges are present
	music	1=yes, 0=no	music in background
	text overlay	1=yes, 0=no	text used during video (pop up, overlay etc, including "comments")
	humor	1=yes, 0=no	Uses humor in the video (makes jokes, does impression etc). Remember it is not about whether you personally think it is funny or not. If the creator is trying to be funny, or make jokes then that is a yes.
	infographic	1=yes, 0=no	host uses chart or graph to display information
	video_style	1=talking to camera, 2=role play/acting, 3=dance, 4=food no host, 5=other no host	Overall style of video, choose most prominent. Select best fit, one only. For example Terry Simpson would usually be a #1, guy impersonating greek grandma would be a #2, #4 refers to videos without a clear host showing **food**, stores, making a recipe with no host (just hands), restaurant etc. #5 is all other content with NO CLEAR HOST. SELECT ONE ONLY
	recipe	1=yes, 0=no	Select yes if the video demonstrates food preparation / recipe. Content should offer instructions, not just mention a dish
	main_message	1=health, 2=culture, 3=other	All videos should fall into one of these categories, select the one that is the closest and select one only. Any health or wellness content should be marked 1, pretty much everything else should be marked 2 unless it has nothing to do with MD and is mistagged. Keep in mind we are interested in the overall main message from the perspective of the poster. That is, what is the creator of the content mainly trying to communicate. Those with both culture and health information should be marked 1. SELECT ONE ONLY

	inaccuracy	1=yes, 0=no	Mark yes if the video appears to contain misinformation, disinformation, inaccurate content EVEN IF unintentional on the part of the creator. For example, red haired lady claims to eat MD, but then orders lamb on white pita bread, since refined grains and red meat are not on the MD, this should be marked yes for inaccuracy
	postsell_product_service	1=yes, 0=no	The video is directly selling a product. This can be done by requesting DMs or offering links to product. This is different from the sales that occur on the poster's information page. For this reason, most of terry simpson's videos would be NO, unless he promotes a specific product or service in the course of an individual post. Also, this is different from a more general promotion (below), wherein someone shares a specific restaurant or product that they like but do not attempt to sell it directly
	promotion	1=yes, 0=no	Post is showing or promoting a specific restaurant, service etc by showing the restaurant name, talking about how great it is etc. This should not include direct sales as noted above.
	unhealthy_foods	0=none, 1=sweets, 2=red meat, 3=refined grains, 4= deepfried foods, 5= ultraprocessed foods	If post promotes unhealthy foods such as baklava, lamb gyro, white pita bread, etc. Do NOT mark if the host is using these foods as an example of what NOT to eat (ie terry simpson pointing out the calories in doritos)
	video_structure	1= original content, 2=stitch, 3=response to comment, 4=other	Type of post, choose one only. Stitch refers to a side-by-side with another video; response to comment is when a comment is clearly visible and host is responding; most others will be original content. If a stitch or comment is shown at any point in the video it should be coded as 2 or 3 respectively. SELECT ONE ONLY
	define	1=yes, 0=no	Yes if a definition of the MD provided at all
	definition	Text	Definition of Mediterranean diet provided (summarize)
	nutrient_info	0=none of these, 1=protein, 2=fat, 3=carbohydrates, 4=fiber, 5=sugar, 6=antioxidants, 7=carotenoids, 8= polyphenols	If any nutrients are mentioned over the course of the post. They need to SAID or SHOWN in CLEAR TEXT on the screen.
	calorie_mention	1=yes, 0=no	If any mention (that is SAID OR SHOWN IN TEXT ON SCREEN) of calories, calorie density, nutrient density or energy density
	anecdote	1=yes, 0=no	Host gives a personal anecdote or story such as losing weight on the MD
	conspiracy	1=yes, 0=no	Organized effort to decieve or share unsubstantiated claims, such as when a host claims that "doctors do not want you to know" about something
	disease_mention	0= none, 1=heart disease, 2= stroke, 3=cancer, 4=diabetes, 5=alzhiemers, 6= other	Specific diseases mentioned in post, for heart disease include similar (cardiovascular disease, hypertension) and also select "other".
	other_CVD_Risk	0=none, 1= blood pressure, 2=weight/obesity, 3=cholesterol/lipids, 4=insulin resistance, 5 = other	Specific cardiometabolic risk factors mentioned in post. These should be clearly mentioned, not inferred.
	inflammation	1=yes, 0=no	Any mention of inflammation, inflammatory biomarkers, anti-inflammatory effects
Post Content Specific to MedDiet	disclaimer	1=yes, 0=no	Disclaimer noted on post clarifying content is only for entertainment, that one should consult their doctor or otherwise
	promotediet	0 = none, 1=low fat, 2=low carb, 3=ketogenic/keto, 4= paleo, 5= DASH, 6=gluten free, 7=other	Type of diet other than Mediterranean diet promoted
	lanuage_english	1=yes, 0=no	Primary language of video is english, all other languages are zero, if langauge is not a factor (because no text/vocals), select 1
	Doctor	1=yes, 0=no	Mention MD is recommended by healthcare providers, or video shows a healthcare provider recommending the MD
	Outside_Food	1=yes, 0=no	Post includes restaurant, takeaway or catered food
	MDS	1=yes, 0=no	Mention MD score or point system, any mention of measuring adherence to MD;
	Study	1=yes, 0=no	Quote health statistics or studies in general such as "scientists have found…", or shows specific studies
	Healthy_General	1=yes, 0=no	Mention MD as healthy diet in general terms including "healthy", "good for you", "best diet" etc.
	MD_Fish	1=yes, 0=no	Any mention of fish or fish clearly shown
	MD_OliveOil	1=yes, 0=no	Any mention of olive oil or olive oil clearly shown. Do not assume a vegetable oil without a label or explanation is olive oil. It needs to be very clear that it is olive oil.
	MD_ModAlcohol	1=yes, 0=no	Any mention of moderating alcohol consumption

MD_FruitVeg	1=yes, 0=no	Any mention of fruit or vegetables (f/v) or f/v clearly shown, so for example- showing greek salad is a yes
MD_WholeGrains	1=yes, 0=no	Any mention of whole grains or whole grains clearly shown (this does NOT include white pita bread, white pasta or other refined grains). It needs to be clear this is WHOLE grains only
MD_Poultry	1=yes, 0=no	Any mention of chicken/turkey/other poultry or poultry clearly shown
MD_LessAnimalProducts	1=yes, 0=no	Any mention of REDUCING meat, dairy or animal fat consumption
MD_Wine	1=yes, 0=no	Any mention of consuming red wine or red wine clearly shown
MD_NutsorBeans	1=yes, 0=no	Any mention of consuming nuts or nuts clearly shown, including peanut butter
MD_Other	open text	Mention of other foods as explicitly being part of the MD, write exactly
Culture_Region_Open	open text	Mention of a specific country, region or cultural aspect related to MD. For example, firstdategirl may mention "Ramadan" "Halal" or "Palestine" and all of these should be open text written in this space
Comments	open text	If you are confused on what to do/it is a hard video to code, use this space to note the column/row and add a comment on what you think is wrong/happening with this
